# Fluid overload-associated large B-cell lymphoma: two case report and review of literature

**DOI:** 10.3389/fonc.2025.1724247

**Published:** 2025-11-26

**Authors:** Liudi Chang, Zhaobo Yang, Cuicui Yang, Yuxiao Wang, Dan Li, Hui Wang, Lanlan Zang, Yuanyuan Zhang

**Affiliations:** 1 School of Clinical Medicine, Shandong Second Medical University, Weifang, China; 2 Department of Hematology, Linyi People’s Hospital, Shandong Second Medical University, Linyi, Shandong, China; 3 Spine surgery, Linyi People’s Hospital, Shandong Second Medical University, Linyi, Shandong, China; 4 Department of Pathology, Linyi People’s Hospital, Shandong Second Medical University, Linyi, Shandong, China; 5 Pharmaceutical laboratory, Department of Pharmacy, Linyi People’s Hospital, Shandong Second Medical University, Linyi, Shandong, China

**Keywords:** lymphoma, fluid overload-associated large B-cell lymphoma, HHV-8 infection, prognosis factor, primary effusion lymphoma

## Abstract

**Background:**

Fluid overload-associated large B-cell lymphoma (FO-LBCL) is an exceptionally rare lymphoma characterized by predominant involvement of serous body cavities—such as the pleura, peritoneum, and pericardium—in the absence of a solid tumor mass. Its low incidence and nonspecific clinical presentation, which often includes symptoms like dyspnea due to effusion, contribute to diagnostic challenges in early stages. This study aims to address current gaps in the understanding of FO-LBCL by reporting two new cases and reviewing the clinical features, treatment regimens, and outcomes of 57 documented patients. Furthermore, through a detailed analysis of FO-LBCL characteristics, this work discusses relevant differential diagnoses and potential treatment strategies.

**Methods:**

A literature search of PubMed and Web of Science was performed using the following search queries: (1) “Fluid overload-associated large B-cell lymphoma” OR “FO-LBC”; (2) “Human herpesvirus 8-unrelated” AND “effusion lymphoma”; (3) “HHV8-unrelated” AND “effusion lymphoma”.

**Results:**

This study included a total of 57 patients. Fluid accumulation most commonly affected the pleural cavity (84.2%), followed by the pericardial (31.6%) and peritoneal (21.1%) cavities. The predominant clinical manifestation was dyspnea (55.8%). Chemotherapy was the primary treatment modality (56.1%), with the R-CHOP regimen representing the most commonly administered protocol. CD20 expression was the most significant favorable prognostic factor (P = 6×10-7). Other factors associated with improved survival included the absence of CD138 expression (P = 0.0009), age ≥ 65 years (P = 0.0015), LDH ≤ 500 U/L (P = 0.0064), the presence of pleural effusion (P = 0.0099), and CD79a expression (P = 0.0411). Treatment with rituximabcontaining chemotherapy regimens was also a significant favorable factor (P = 0.0036).

**Conclusions:**

FO-LBCL often presents with dyspnea caused by fluid effusion. Routine laboratory tests typically show no significant abnormalities, making timely pathological examination essential for a definitive diagnosis. Clinicians should enhance their understanding of FO-LBCL characteristics to improve early diagnostic accuracy. It is crucial to select appropriate treatment strategies based on prognostic factors.

## 1 Introduction

Fluid overload–associated large B-cell lymphoma (FO-LBCL) is a rare subtype of large B-cell lymphoma characterized by selective involvement of serous body cavities (pleura, pericardium, and peritoneum) without formation of a palpable solid mass. Current epidemiological estimates indicate that FO-LBCL accounts for <0.1% of lymphoid malignancies; among reported cases, approximately 60% have been described in Japanese patients. FO-LBCL is not associated with Kaposi sarcoma–associated herpesvirus (KSHV)/human herpesvirus 8 (HHV-8) infection. Although numerous HHV-8–unrelated effusion lymphomas were previously reported, they were not clearly defined in early lymphoma classifications. HHV-8–positive effusion lymphoma was historically designated primary effusion lymphoma (PEL), while KSHV/HHV-8–negative effusion lymphomas were variously described as PEL variants, PEL-like lymphomas, or KSHV/HHV-8–unrelated B-cell proliferative disorders. FO-LBCL was formally recognized as a distinct entity within large B-cell lymphomas in the 2022 WHO 5th Edition Classification of Tumours of Haematolymphoid Tissues. Clinically, patients with FO-LBCL commonly present with dyspnea secondary to serous effusion, while routine laboratory investigations are frequently nondiagnostic, which complicates and delays definitive diagnosis. Here, we report two cases presenting with severe dyspnea attributable to pericardial and pleural effusions, respectively, and we review the clinical features, treatment regimens, and outcomes of 57 FO-LBCL patients to characterize disease manifestations and inform differential diagnosis and management strategies.

## 2 Materials and methods

### 2.1 Data retrieval and methodology

**Figure 1:**
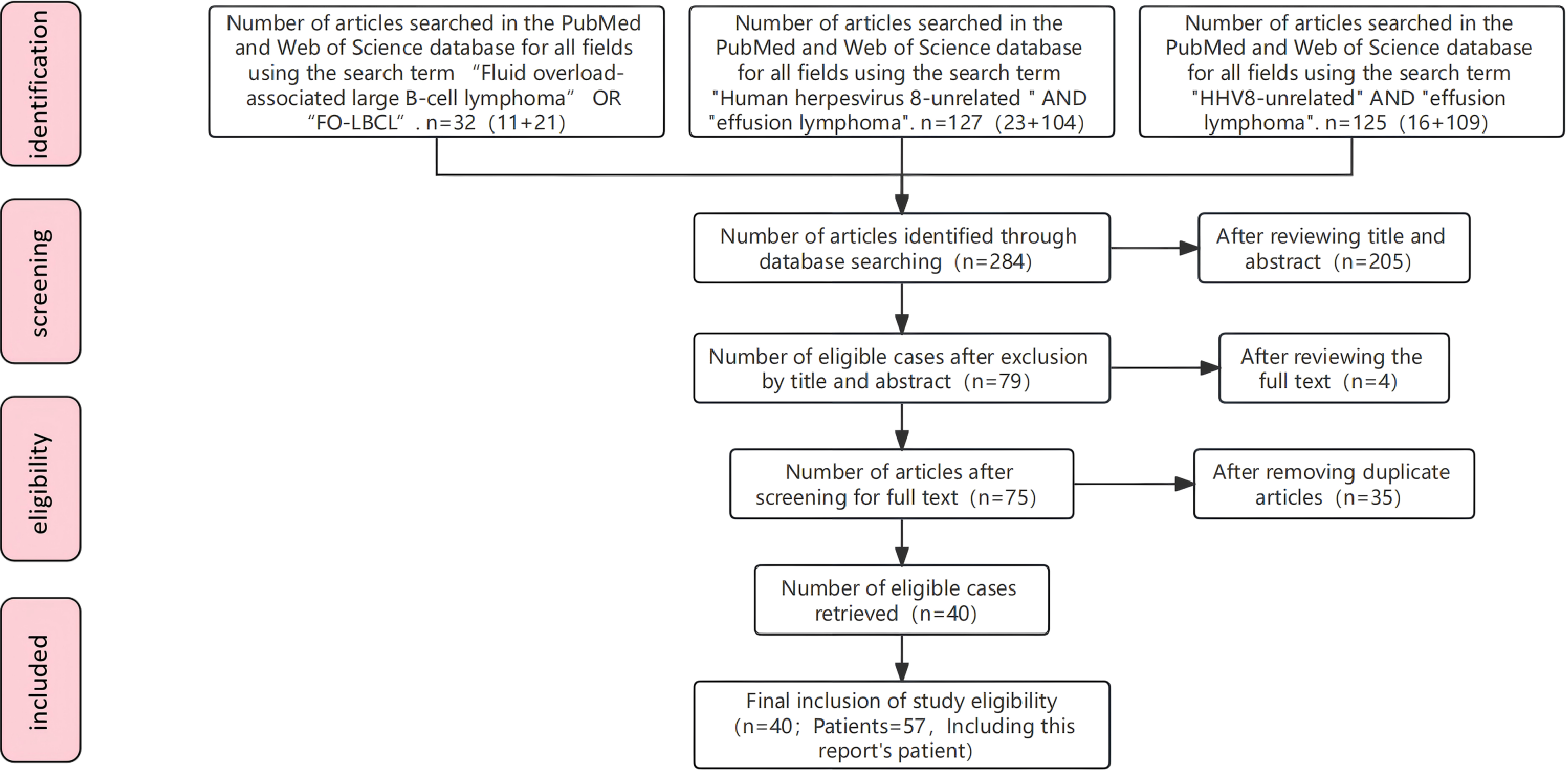
Flow chart depicting the systematic review process utilized in this study.

A literature search of PubMed and Web of Science was performed using the following search queries: (1) “Fluid overload-associated large B-cell lymphoma” OR “FO-LBC”; (2) “Human herpesvirus 8-unrelated” AND “effusion lymphoma”; (3) “HHV8-unrelated” AND “effusion lymphoma”. A total of 283 articles were retrieved. All titles, abstracts, and full texts were screened according to the “inclusion criteria” described below. Ultimately, 55 patients reported in 40 papers were included. Combined with the 2 patients reported in this study, a total of 57 patients were enrolled (**Figure 1**).

The inclusion criteria were as follows (1–3): (1) Patients in whom serous effusion (confirmed by paracentesis, thoracentesis, or pericardiocentesis) was the initial manifestation of disease, and who had no imaging or clinical evidence of lymphoma-related lymphadenopathy or concomitant solid tumors, were included; (2) The absence of HHV-8/KSHV infection can be confirmed through several methods, including immunohistochemistry for latent nuclear antigen-1, polymerase chain reaction (PCR) for viral DNA in effusion or tissue specimens, or *in situ* hybridization for viral genomic DNA within tumor cells. Alternatively, a negative status can be considered confirmed if explicitly stated in the original literature, even if the specific detection methodology is not detailed. (3) exclusion of alternative diagnoses, specifically primary effusion lymphoma, empyema-associated lymphoma, and diffuse large B-cell lymphoma presenting with malignant body-cavity effusion; (4) no restrictions on age or sex; and (5) for duplicated reports, only the earliest published case was included.

### 2.2 Statistical analysis

Statistical analyses were performed using SPSS version 27.0 and GraphPad Prism version 10.0. Continuous variables are reported as mean ± standard deviation or median (interquartile range), and categorical variables as counts and percentages. Overall survival (OS) was defined as the interval from diagnosis to death from any cause or to the date of last follow-up. For cases in which a definitive diagnosis at presentation was delayed because of insufficient diagnostic evidence, OS was calculated from the initiation of antitumor therapy. For patients who remained alive after multiple chemotherapy cycles but lacked a clearly recorded date of last follow-up, follow-up time was estimated from the number and type of chemotherapy cycles received. The estimation rules were: each cycle of rituximab monotherapy was counted as 1 week, and each cycle of R-CHOP (rituximab, cyclophosphamide, doxorubicin, vincristine, and prednisolone) or CHOP was counted as 3 weeks. Survival curves were generated using the Kaplan–Meier method and compared by the log-rank test. Cox proportional hazards regression was used to analyze overall survival (OS) and to identify independent prognostic factors.

## 3 Results

A total of 57 patients meeting the inclusion criteria was enrolled, with a male-to-female ratio of 2:1 (38 males, 66.7%; 19 females, 33.3%). The age range was 29 to 99 years, with a median age of 75. The pleural cavity was the most frequent site of fluid involvement (48/57, 84.2%), followed by the pericardial (18/57, 31.6%) and peritoneal (12/57, 21.1%) cavities. Consistent with the predominant pleural involvement, dyspnea was the most common presenting symptom, reported in 55.8% (24/43) of patients. Other reported symptoms included chest tightness, abdominal discomfort, fatigue, and lower limb edema, reflecting multisystem involvement. Most patients had comorbidities associated with chronic fluid overload, including liver cirrhosis, heart failure, and chronic kidney disease (**Table 1**). Consistent with prior literature, FO-LBCL occurred predominantly in HIV-negative individuals. The neoplastic cells typically expressed pan-B-cell markers and were negative for CD3, CD5, CD10, CD30, and CD138. Therapeutic data were available for all 57 patients. A total of 32 patients (56.1%) received chemotherapy, while the remaining 25 (43.9%) did not. Among the 32 patients who received chemotherapy, the majority (n=17) were treated with a rituximab-containing CHOP-like regimen. Other rituximab-based therapies were used in four patients (two with monotherapy and two with other regimens). The remaining eight patients received regimens without rituximab, comprising six who received a CHOP-like regimen and two on a non-CHOP-like regimen. Chemotherapy details were unavailable for three patients. Survival data were available for 49 patients, with a median overall survival of 10.5 months. The disease-related mortality rate was lower among patients who received chemotherapy (30.0%, 9/30) compared to those who did not (36.8%, 7/19) (**Table 2**).

**Table 1:** Chart of 57 patients with fluid overload-associated large B-cell lymphoma.

Case	Age/ Gender	Other disease	Involved sites	Cell morphology	Immunophenotype	Therapy	Final outcome	Follow-up (months)
1 (4)	90/F	Hypertension	Pericardial cavity、Bilateral pleural cavities		CD20、PAX-5、BCL-2、MUM1	Drainage	Death	3
2 (4)	83/F	T2DM	Pericardial cavity、Bilateral pleural cavities		CD20、PAX-5、BCL-6、MUM1	Drainage	Death	18
3 (4)	71/M	Hypertension、T2DM	Pericardial cavity、Bilateral pleural cavities		CD20、PAX-5、BCL-6(Rare)	Drainage	Alive	10
4 (5)	72/M	UC	Peritoneal cavity	Large	CD79a、CD20、BCL-2、BCL6、MUM1	Drainage+CHOP×6	Death	7
5 (6)	66/M		Bilateral pleural cavities	Medium-to-large	CD20、PAX5、BCL-6、CD10、MUM1	Drainage	Alive	24
6 (7)	82/F	Hypertension、Af、CKD	Left pleural cavity	Medium-to-large	CD20、MUM1、BCL2、BCL6(Weak Expression)	R-mini-CHOP×6	Alive	4.5
7 (7)	59/F	Hypertension	Pericardial cavity	Medium-to-large	CD20、BCL6	Drainage	Alive	NA
8 (8)	80/M	Hypertension、Af	Right pleural cavity	Large	CD20、CD79a、BCL-2、BCL-6	Drainage+R-THP-COP	Death	13
9 (9)	82/M	Hypertension、Af、T2DM、CAD	Right pleural cavity	Large	CD45、CD20、PAX-5、MUM-1、BCL6、BCL2	Drainage	NA	NA
10 (10)	69/M		Bilateral pleural cavities	Large	CD10、CD20、CD79a、PAX5、MUM1、BCL-2、BCL-6	Drainage	Alive	22
11 (**2**)	70/M	Liver cirrhosis	Peritoneal cavity	Large	CD20、CD79a、PAX5、CD3、CD19、BCL-6、LCA	Drainage+ chemotherapy	Alive	NA
12 (11)	72/F		Left pleural cavity、Pericardial cavity	Large	CD20、CD38、CD10、BCL-2、BCL-6-MUM1	EPOCH-R×6	Alive	10.5
13 (12)	79/M	Hypertension、Aortic dissection	Left pleural cavity	Large	CD45、CD20、CD79a、BCL-2、BCL-6、MUM1	Pleural fixation	Alive	55
14 (13)	70/M	Heart disease	Pleural cavities		CD20	R + SOB + ETO	Alive	7.75
15 (14)	71/F		Pericardial cavity、Bilateral pleural cavities、Peritoneal cavity		CD20、CD79	Drainage+ chemotherapy	NA	NA
16 (15)	89/F		Left pleural cavity	Large	CD19、CD20、CD79a、CD10、CD38、CD7、BCL-2、BCL-6	ETO-mono +R-mono	Death	13
17 (16)	72/M	Esophageal Cancer	Bilateral pleural cavities、Pericardial cavity	Medium-to-large	CD45、CD20、CD79a、PAX5(Weak Expression)、CD30(Scattered Expression)	Drainage+CHOP×1.5	Alive	23.13
18 (17)	69/M	CI、CML	Bilateral pleural cavities	Medium-to-large, pleomorphic	CD20、CD79a、CD99、BCL-2、BCL-6、MUM1	R-CHOP×2+R-CVP×3	Alive	3.75
19 (18)	79/M	MDS、Heart disease	Peritoneal cavity	Large	CD20、BCL-2、BCL-6	Drainage	NA	NA
20 (19)	89/M	Hypertension、DM、CAD	Right pleural cavity		CD45、CD30、CD38、CD71	None	Alive	40
21 (20)	55/M	Liver cirrhosis	Right pleural cavity、Peritoneal cavity	Large	CD20、CD79a、BCL-6、MUM1、BCL-2	R2×8+ LEN	Death	20
22 (21)	80/F	Hypertension	Left pleural cavity	Medium-to-large	CD20、BCL-2	None	Alive	18
23 (22)	99/F		Pericardial cavity、Bilateral pleural cavities	Medium-to-large	CD20、CD5、CD19、CD20、CD25	Drainage	Death	16
24 (22)	85/M	Hypertension、Af	Pericardial cavity、Bilateral pleural cavities	Medium-to-large	CD20	Drainage	Alive	11
25 (23)	64/F		Right pleural cavity	Large	CD10、CD19、CD20、CD30、CD38	Drainage+CHOP×1	Alive	12
26 (24)	65/F		Bilateral pleural cavities	Large	CD20、CD79a、MUM1、CD138、PAX-5	R-CHOP×3	Death	5.5
27 (25)	63/M	Hypertension、DM	Pericardial cavity、Bilateral pleural cavities、Peritoneal cavity	Large	CD20、CD79	R-CHOP×6, CHASER×1	Alive	37.25
28 (25)	84/M	DM、CHF	Pericardial cavity、Left pleural cavity	Large	CD20、CD79	Drainage	Death	16
29 (25)	78/F		Left pleural cavity	Large	CD20、CD79	Drainage+ R-mono×8	Alive	25
30 (25)	89/M	Hypertension	Right pleural cavity	Large	CD10、CD19、CD45	Drainage	Death	0.5
31 (26)	79/M	Hypertension、Colon cancer、DM	Left pleural cavity	Medium-to-large	CD20、CD79a	R-THP-COP×2	Alive	7.5
32 (27)	73/M		Pericardial cavity	Large	CD20、CD79a、MUM1、PAX-5(Partial Expression)	R-CHOP×2+radiotherapy	Death	3
33 (28)	76/M	EC	Right pleural cavity	Large	CD19、CD20、CD30、CD79a	R-CHOP×6	Alive	22.5
34 (29)	85/M		Pericardial cavity、Bilateral pleural cavities	Medium-to-large	CD45、CD20、BCL-2、CD30、MUM1	Drainage	Alive	24
35 (30)	77/M		Right pleural cavity、Peritoneal cavity	Large, pleomorphic	CD45、CD20、MUM1	R-CHOP×6	Alive	19.5
36 (31)	73/F		Pleural cavities	Pleomorphic	CD45、CD20、CD79a	R-CHOP×6	Death	112.5
37 (32)	75/M	CAD、Af、CHF	Bilateral pleural cavities		CD20、CD30(Weak Expression)、CD45、MUM1、BCL-6	None	NA	NA
38 (33)	82/M		Pericardial cavity、Left pleural cavity	Medium-to-large	CD20、CD79a	Drainage+R-CHOP×6	Alive	16.5
39 (33)	73/M		Pericardial cavity、Pleural cavities、Peritoneal cavity	Large	CD20	Drainage+R-CHOP×6	Alive	4.5
40 (3)	75/M	Hypertension、T2DM	Pericardial cavity、Left pleural cavity			EPOCH×2	Alive	1.5
41 (34)	44/F	DM、Cholecystitis、Spinal osteoarthritis	Bilateral pleural cavities、Peritoneal cavity	Large, pleomorphic	CD45、CD10、CD38	CHOP×2	Death	2
42 (5)	88/F		Left pleural cavity	Pleomorphic	CD38、CD45、CD138、MUM1	Drainage	Death	11
43 (5)	85/F	Endometrial cancer	Right pleural cavity	Pleomorphic	CD45、cyclinD1、CD138、c-myc	Drainage+ chemotherapy	Death	1.5
44 (35)	85/F	CAD	Left pleural cavity	Large, pleomorphic	CD45, CD20, CD79a, CD138, BOB1, OCT2, BCL6, MUM1	Drainage	lost to follow-up	NA
45 (35)	29/M	CHD	Peritoneal cavity	Large, pleomorphic	CD45、CD38、CD79a、CD138、PAX5、BOB1、OCT2、KLC	ICE+BCNU-ECM+SCT	Death	4.5
46 (35)	45/M	ALC	Peritoneal cavity	Large, pleomorphic	CD45、CD38、CD56、CD138	CHOP×1	Death	1.5
47 (35)	72/M	CAD	Pleural cavity	Large, pleomorphic	CD45、CD20、CD30、CD43、BCL2	Drainage	lost to follow-up	NA
48 (35)	51/M	ALC	Bilateral pleural cavities	Large, pleomorphic	CD45、CD25、CD30、CD38、MUM1	Drainage	Death	0.03
49 (36)	75/M	Hypertension、Tuberculosis	Pleural cavity	Large	CD45、CD20、CD79a、PAX5	Drainage	Alive	13
50 (37)	55/M	ALC	Right pleural cavity	Large, pleomorphic		Drainage	Death	4
51 (37)	95/M	CAD、Af	Left pleural cavity	Medium-to-large	CD19、CD20、CD22、CD10	R-mono	Alive	72
52 (37)	69/F	Thyroid cancer、Biliary pancreatitis	Pericardial cavity	Medium-to-large, pleomorphic	CD19、CD20、CD38	R-CHOP×6	Alive	4.5
53 (38)	61/M	ALC	Left pleural cavity、Peritoneal cavity	Large, pleomorphic	CD138、EMA、MUM1	No chemotherapy	Death	0.5
54 (39)	44/M		Right pleural cavity		CD3、CD16、CD8	None	Death	0.03
55 (40)	77/M	CAD、Prostate cancer	Right pleural cavity	Large	CD19、CD20、cCD79a、CD38、CD66、CD71、CD45、 CD30、EMA	R-CHOP	Alive	3
56	73/F		Pericardial cavity	Medium-to-large	CD20、CD79a、LCA	Drainage+R-COP×6+R-CMOP×1	Alive	34
57	75/M		Right pleural cavity	Medium-to-large	CD2、CD79a、LCA	R-CHOP×2	Alive	6

F, female; M, male; DM, diabetes mellitus; T2DM, Type 2 diabetes mellitus; UC, ulcerative colitis; Af, Atrial fibrillation; CKD, Chronic Kidney Disease; CAD, Coronary Artery Disease; CI, Cerebral Infarction; CML, Chronic myelocytic leukemia; MDS, Myelodysplastic syndromes; CHF, Chronic heart failure; EC, Esophageal cancer; CHD, Congenital Heart Disease; ALC, Alcoholic liver cirrhosis; R/R-mono, Rituximab; R-CHOP, Cyclophosphamide + Doxorubicin + Vincristine + Prednisone; EPOCH, Etoposide + Prednisone + Vincristine + Cyclophosphamide + Doxorubicin; SOB, sobuzoxane; ETO/ETO-mono, etoposide; R-CVP, Rituximab + Cyclophosphamide + Vincristine + Prednisolone; R2, Rituximab + Lenalidomide; LEN, Lenalidomide; CHSAER, Rituximab + Cyclophosphamide + Cytarabine + Etoposide + Dexamethasone; R-THP-COP, Rituximab + Pirarubicin + Cyclophosphamide + Vincristine + Prednisolone; ICE, Ifosfamide + Carboplatin + Etoposide; BCNU-ECM, BischloroethylNitrosUrea (Carmustine) + Etoposide + Cyclophosphamide + Mesna; SCT, Stem Cell Transplantation; CMOP, Cyclophosphamide + Mitoxantrone + Oncovin + Prednisone; “NA” means not mentioned in the text; “None” means untreated.

**Table 2:** Characteristics of 57 patients with FO-LBCL.

	Value	%
Age, median years (range), n=57	75(29-99)	
Sex, male/female, n=57	38/19	66.7%/33.3%
Clinical symptoms, n=44		
Dyspnea\​	24	54.5%
Shortness of breath	11	25.0%
Chest tightness/Chest pain	5	11.4%
Abdominal pain/Abdominal distension	6	13.6%
Fatigue	7	15.9%
Lower limb edema	6	13.6%
Cough	7	15.9%
Fever	4	9.1%
Syncope\​	1	2.3%
Type of body cavity involved		
Pleural cavities (+/\−), n=57	48/9	84.2%
Pericardial cavity (+/\−), n=57	18/39	31.6%
Peritoneal cavity (+/\−), n=57	12/45	21.1%
Number of body cavity involved (1/2/3), n=57	39/15/3	68.4%/26.3%/5.3%
Virus		
HHV-8 (+/\−), n=57	0/57	0%
HIV (+/\−), n=39	1/38	2.6%
EBV (+/\−), n=43	10/33	23.3%
HBV (+/\−), n=26	2/24	7.7%
HCV (+/\−), n=37	3/34	8.1%
Cell morphology, n=45		
Medium-to-large	15	33.3%
Large	30	66.7%
Immunophenotype		
CD19 (+/\−), n=11	7/4	63.6%
CD20 (+/\−), n=52	45/7	86.5%
CD79a (+/\−), n=30	24/6	80.0%
CD10 (+/\−), n=27	9/18	33.3%
CD30 (+/\−), n=26	9/17	34.6%
CD138 (+/\−), n=23	7/16	30.4%
Cytogenetic abnormalities(+/\−), n=14	11/3	78.6%
MYC gene re-arrangement, n=15	4/11	26.7%
Treatment, n=57		
No chemotherapy	25	43.9%
Chemotherapy	32	56.1%
R monotherapy	2	
CHOP-like chemotherapy with R	17	
CHOP-like chemotherapy without R	6	
Non-CHOP-like chemotherapy with R	2	
Non-CHOP-like chemotherapy without R	2	
Unknown regimen	3	

R, rituximab; CHOP, Cyclophosphamide + Doxorubicin + Vincristine + Prednisone.

**Figure 2:**
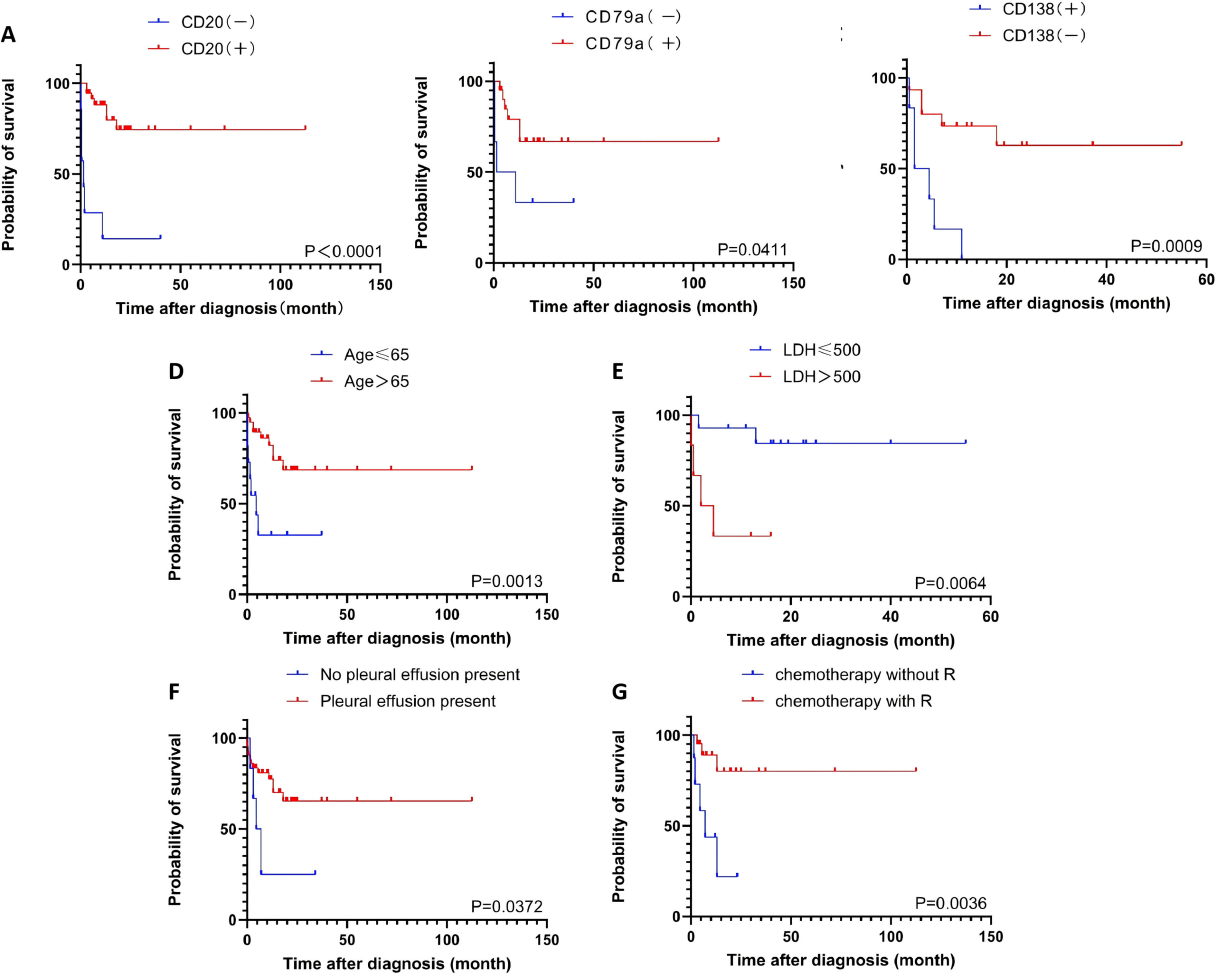
The overall survival according to the CD20 **(A)**, the CD79a **(B)**, the CD138 **(C)**, the age **(D)**, the LDH level **(E)**, the pleural effusion **(F)**, the chemotherapy regimen **(G)**.

Univariate analysis of overall survival was performed on cases with complete prognostic and clinical data. CD20 expression was the most significant favorable prognostic factor (P = 6×10^-7^). Other factors associated with improved survival included the absence of CD138 expression (P = 0.0009), age ≥65 years (P = 0.0015), LDH ≤500 U/L (P = 0.0064), the presence of pleural effusion (P = 0.0099), and CD79a expression (P=0.0411). Treatment with rituximab-containing chemotherapy regimens was also a significant favorable factor (P=0.0036) (**Table 3**; **Figure 2**).

**Table 3:** Univariate analysis for overall survival.

Variables	n	Ρ value
Age		
≥65 years	11	0.0013
<65 years	38	
Sex		
Male	33	0.4035
Female	16	
Number of body cavity involved		
1 cavity	32	0.3206
≥2 cavities	17	
Type of body cavity involved		
Pleura (+/−)	43/6	0.072
Pericardia (+/−)	16/33	0.1682
Peritoneum (+/−)	9/40	0.0945
Virus		
HIV (+/−)	1/35	0.0679
EBV (+/−)	10/28	0.2760
HCV (+/−)	3/29	0.2719
HBV (+/−)	2/20	0.5799
Immunophenotype		
CD19 (+/−)	7/4	0.1854
CD20 (+/−)	37/7	<0.0001
CD79a (+/−)	21/6	0.0411
CD10 (+/−)	8/15	0.8183
CD30 (+/−)	7/17	0.1589
CD138 (+/−)	6/15	0.0009
Serum LDH level		
≤250 IU/L	0	0.0807
>250 IU/L	20	
≤500 IU/L	14	0.0064
>500 IU/L	6	
MYC gene e-arrangement (+/−)	4/10	0.6459
Therapy		
Chemotherapy with R	8	0.0036
Chemotherapy without R	21	

LDH, the serum lactate dehydrogenase.

## 4 Case report

### Case 1

**Figure 3:**
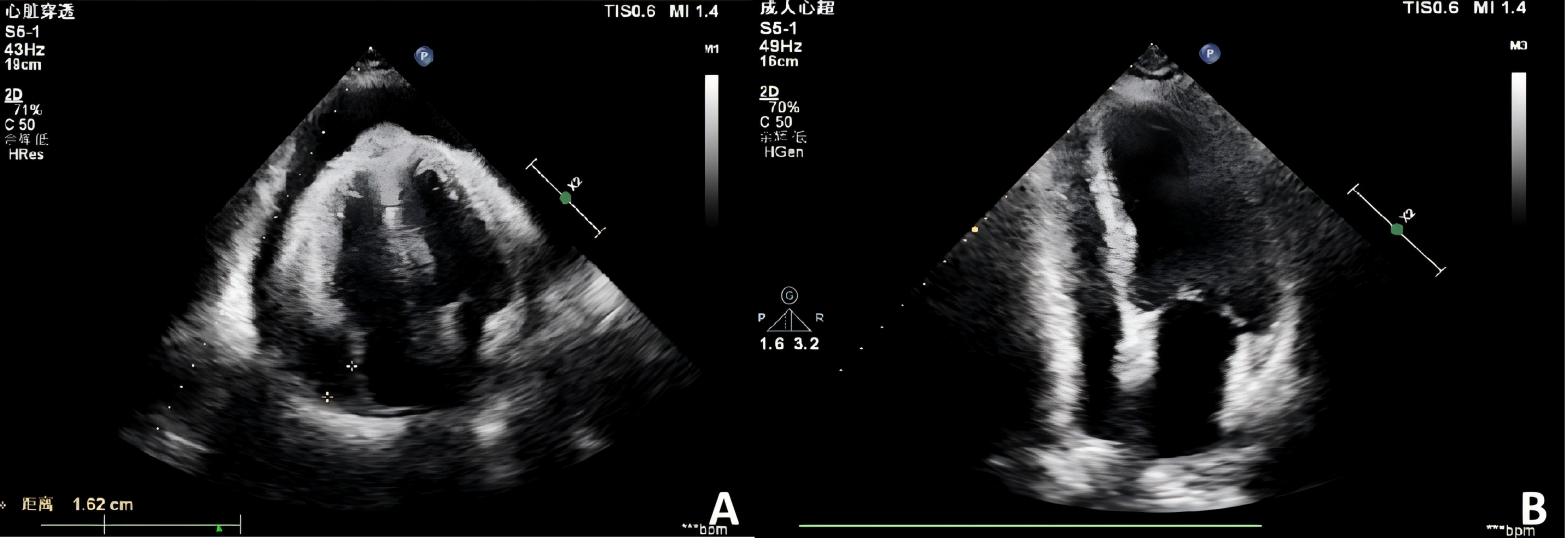
Echocardiographic assessment of pericardial effusion. **(A)** Initial echocardiogram (October 2022) reveals a moderate to severe pericardial effusion. **(B)** Follow-up study (June 2024) demonstrates complete resolution of the effusion.

**Figure 4:**
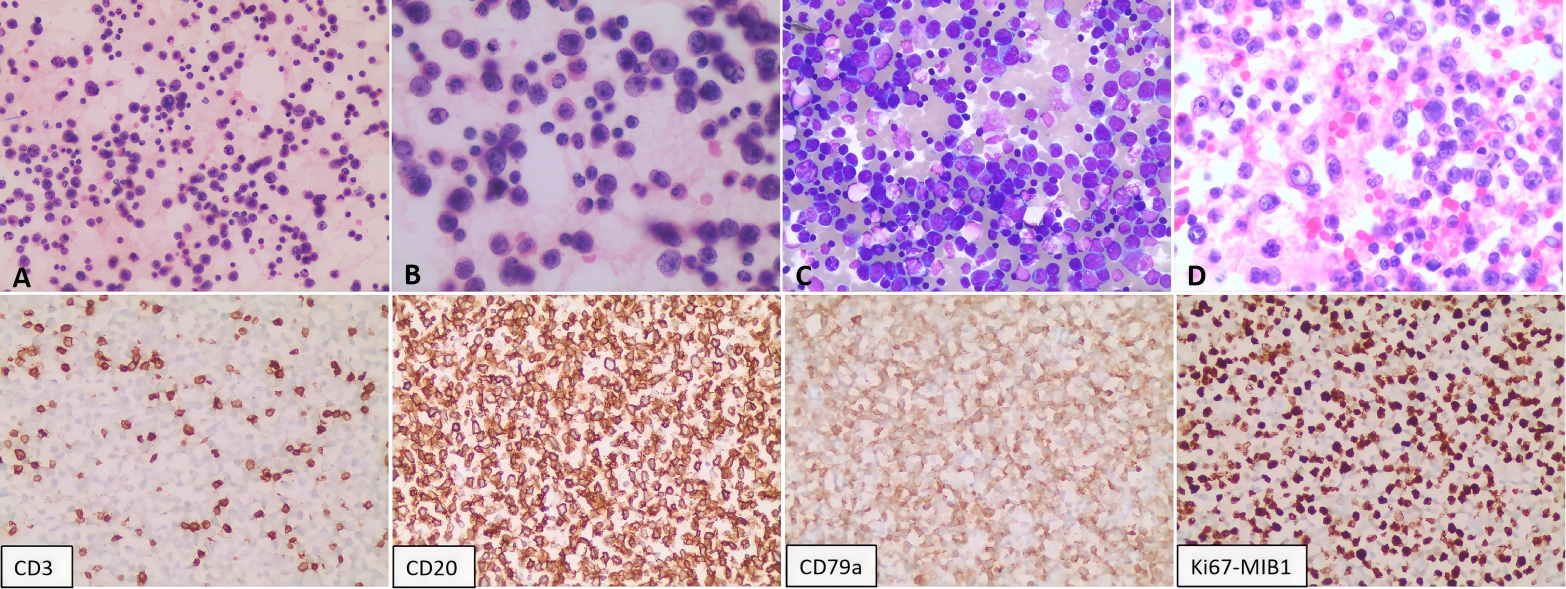
Pathological analysis of the pericardial effusion. Cytological smears show diffuse, scattered atypical lymphoid cells of moderate to large size. The cells exhibit round-to-oval nuclei, vacuolated chromatin, visible nucleoli, scant cytoplasm, and occasional mitotic figures. **(A, B)** Hematoxylin and eosin (H&E) staining at 200X and 400X magnification, respectively. **(C)** Giemsa stain (200X). **(D)** Cell block preparation from the sediment (H&E, 400X). Immunohistochemistry on the cell block specimen was positive for CD20 and CD79a, negative for CD3, and demonstrated a high proliferative index (Ki-67 approximately 70%).

A 73-year-old female presented in October 2022 with a ten-day history of exertional dyspnea. Her medical history was unremarkable. Laboratory investigations—including complete blood count, biochemistry, cardiac enzymes, coagulation studies, and serological tests for tuberculosis, EBV, cytomegalovirus, HHV-8, hepatitis B, hepatitis C, and HIV—were within normal limits. Cardiac ultrasonography revealed a moderate to massive pericardial effusion (**Figure 3A**). Pericardiocentesis was performed, draining approximately 900 ml of bloody fluid, and a cytological specimen was sent for pathological analysis. Microscopic examination revealed small, round tumor cells, suggestive of a hematolymphoid neoplasm of B-cell origin. Immunohistochemical analysis was positive for CD20, LCA, and CD79a, and negative for calretinin, Ber-EP4, cytokeratin (CK), CD3, and TTF-1. The Ki-67 (MIB-1) proliferation index was approximately 70% (**Figure 4**). A diagnosis of B-cell lymphoma was established, and the patient received one cycle of R-COP chemotherapy. A follow-up PET-CT scan on November 21, 2022, demonstrated diffusely heterogeneous increased FDG metabolism in the bone marrow (SUVmax 7.5), a finding suggestive of a myeloproliferative change, potentially treatment-related. No other foci of abnormal uptake indicative of lymphoma were identified. Following the initial cycle, the patient completed five additional cycles of R-COP chemotherapy, for a total of six cycles. Follow-up imaging with bilateral lung and whole-abdominal CT in October 2023 was unremarkable. A subsequent cardiac echocardiography and abdominal CT in June 2024 also showed no abnormalities (**Figure 3B**). Based on these findings, the patient was assessed as having achieved complete remission (CR).

**Figure 5:**
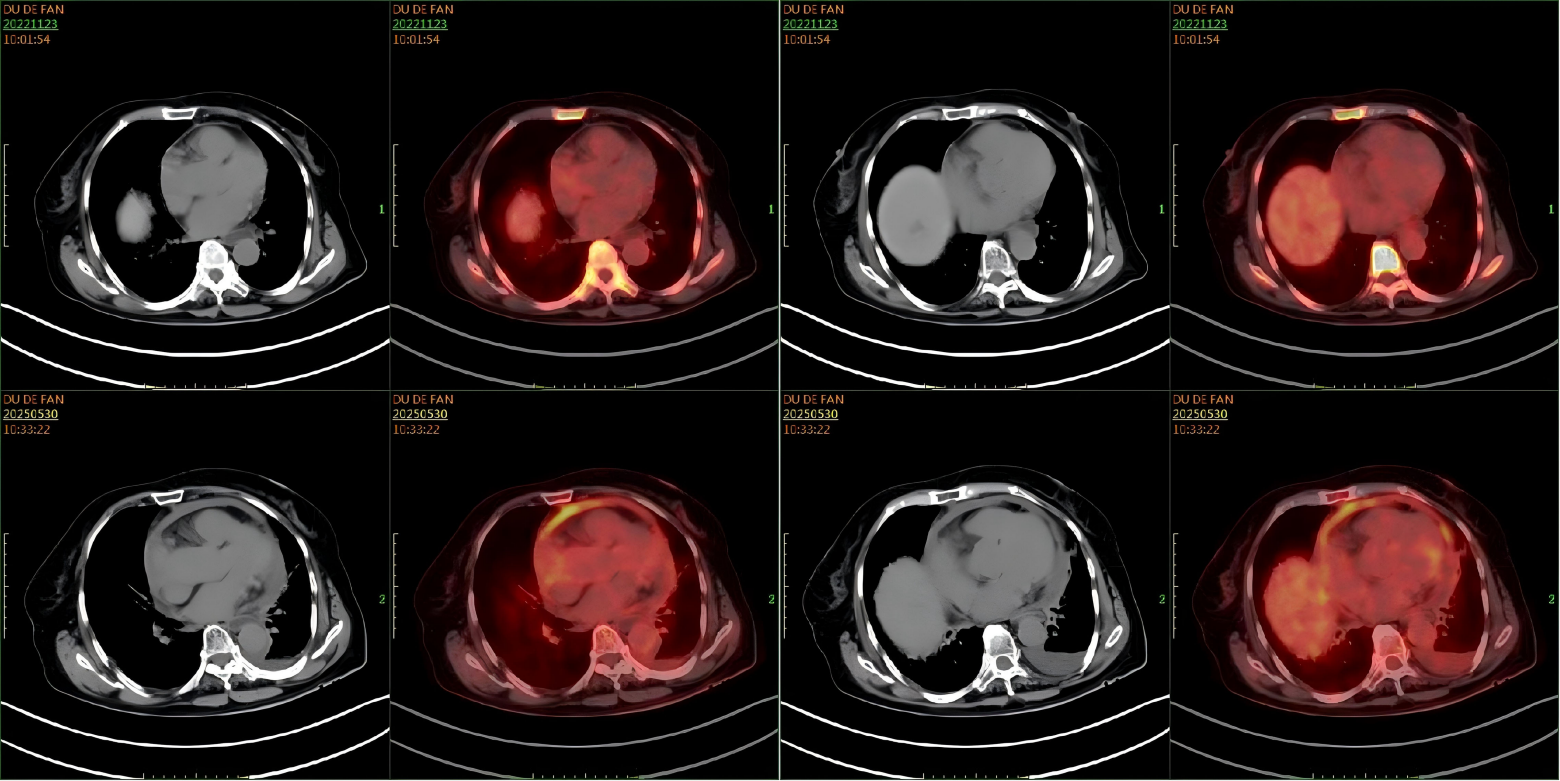
Comparison of PET-CT scans. A comparison of cardiac imaging from November 23, 2022, and May 29, 2025, reveals new findings of pericardial thickening with decreased fluid density, cardiac enlargement, and increased peripheral fluorodeoxyglucose (FDG) uptake.

However, in May 2025, the patient was re-admitted with a two-day history of dyspnea, accompanied by chest tightness, chest pain, and a productive cough with white sputum. She was febrile, with a maximum temperature of 38.0 °C. Laboratory investigations were notable only for mildly elevated inflammatory markers; complete blood count, biochemical panels, lactate dehydrogenase (LDH), and cardiac enzymes were otherwise within normal limits. An electrocardiogram revealed frequent ventricular premature beats in triplets, and cardiac echocardiography confirmed the recurrence of pericardial effusion. However, flow cytometry and histopathological analysis of the effusion were unremarkable. A follow-up PET-CT scan on May 29, 2025, was subsequently performed. Comparison with the scan from November 2022 demonstrated new pericardial thickening and effusion with associated increased FDG uptake showing peripheral enhancement (**Figure 5**). This new finding was highly suggestive of disease recurrence. No other foci indicative of lymphoma were identified. The initial pericardial effusion pathology specimen was subjected to an expert pathological consultation. The differential diagnosis included fluid overload-associated large B-cell lymphoma and diffuse large B-cell lymphoma, not otherwise specified (DLBCL-NOS). Immunohistochemical staining revealed a profile positive for PAX5, with partial positivity for BCL-2 and MUM1, and focal positivity for MYC and P53. The neoplastic cells were negative for CD5, CD10, CD138, and Cyclin D1. The disease was confined to the pericardial effusion, with no evidence of lymphoma involvement at other sites. A final diagnosis of FO-LBCL was established. Due to compromised cardiac function, the chemotherapy regimen was modified to R-CMOP. The patient has completed one cycle of this adjusted regimen and was alive and under ongoing follow-up as of August 2025.

### Case 2

**Figure 6:**
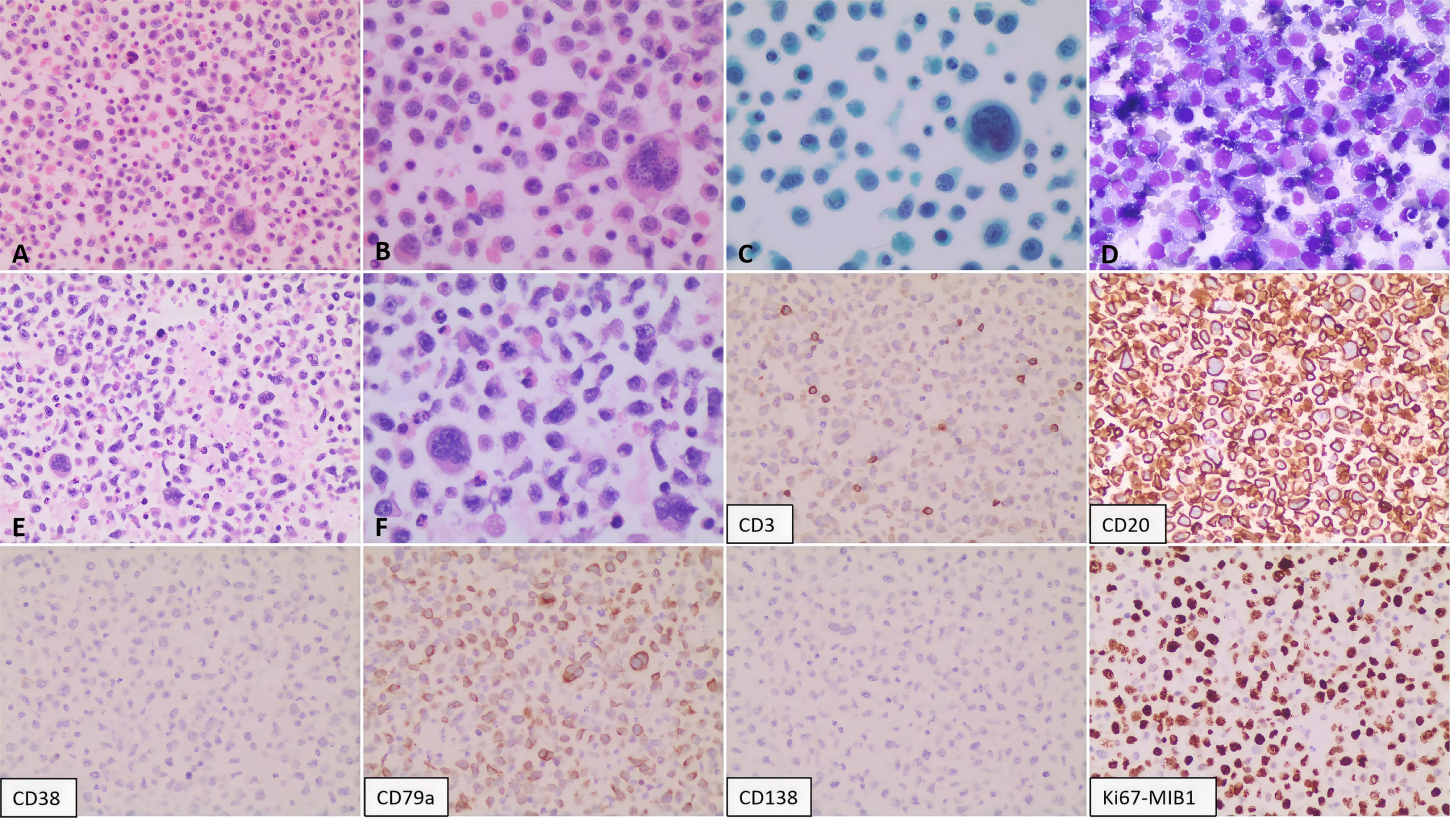
Pathological findings of the pleural effusion. The smear shows a diffuse population of scattered atypical lymphoid cells. These cells are moderately sized and characterized by round to oval nuclei with coarse, granular chromatin; prominent nucleoli are evident in a subset. The cytoplasm is abundant. Mitotic figures and multinucleated giant cells are present. Panels illustrate: **(A, B)** Cytology (H&E stain; 200X and 400X, respectively); **(C)** Cytology (Papanicolaou stain; 400X); **(D)** Cytology (routine stain; 200X); **(E, F)** Sediment cell block sections (H&E stain; 200X and 400X, respectively). Immunohistochemical analysis of the pericardial effusion reveals the following immunophenotype: CD2(+), CD79a (mostly positive), Ki-67 (approximately 80%), with negative staining for CD3, CD38, and CD138.

**Figure 7:**
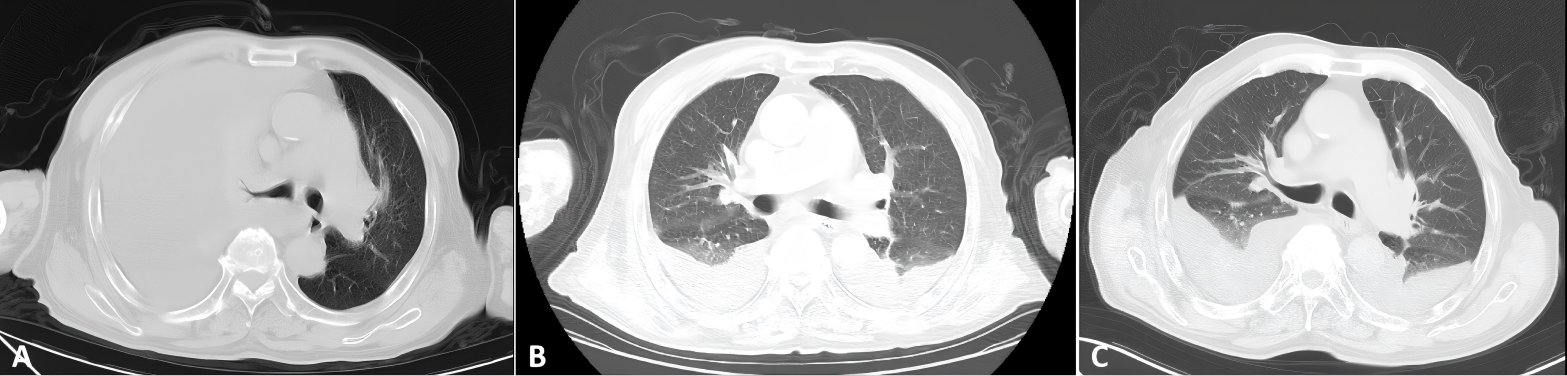
Serial bilateral chest CT imaging of pleural effusion. **(A)** February 2025: Image obtained at initial discovery. **(B)** March 2025: Pre-treatment follow-up scan. **(C)** April 2025: Scan obtained after one cycle of chemotherapy.

A 75-year-old male was admitted in February 2025 with a ten-day history of cough and dyspnea. The patient was otherwise healthy with no significant past medical history. Laboratory results, including complete blood count, biochemistry, cardiac enzymes, coagulation studies, tuberculin T-cell assay, and standard infection markers, were within normal limits. Serological tests for HCV, HBV, HIV, and HHV-8 were negative. Cardiac ultrasonography revealed no abnormalities. However, ultrasonography of the chest identified a massive right pleural effusion, with maximum vertical and horizontal diameters of 224 mm and 158 mm, respectively. Analysis of the pleural fluid demonstrated markedly elevated adenosine deaminase (ADA) at 204.7 U/L and lactate dehydrogenase (LDH) at 2086.1 U/L, while the carcinoembryonic antigen (CEA) level was within normal limits at 1.67 ng/mL. Microbiological and molecular studies for tuberculosis, including Mycobacterium tuberculosis DNA PCR, acid-fast bacillus (AFB) smear, and T-cell-based testing, were negative. Pathological examination of the pleural effusion was suggestive of a hematolymphoid neoplasm, likely of B-cell origin. Immunohistochemistry (**Figure 6**) demonstrated that the neoplastic cells were positive for LCA, CD20, and CD79a (mostly positive), with a high proliferation index (Ki-67 approximately 80%). The cells were negative for Ber-EP4, CEA, CD3, CD38, and CD138. Flow cytometric analysis confirmed a population of abnormal large cells, constituting 63.65% of nucleated cells, with an immunophenotype positive for CD20, CD22, CD81, and CD45, and negative for CD5, CD10, CD3, and CD38. These findings support a final diagnosis of large B-cell lymphoma with pleural involvement. Bone marrow examination, including cytology, biopsy, and flow cytometry, revealed no evidence of lymphomatous involvement. Similarly, a left axillary lymph node fine-needle aspiration biopsy was unremarkable. Based on the integrated clinical presentation and immunophenotypic profile, a definitive diagnosis of FO-LBCL was established. A follow-up bilateral chest CT scan on 6 March 2025 showed bilateral pleural effusions with adjacent pulmonary hyperexpansion and a minimal pericardial effusion (**Figure 7B**). The patient subsequently received two cycles of R-CHOP chemotherapy. However, a follow-up bilateral lung CT scan after the first cycle (**Figure 7C**) indicated a lack of significant therapeutic response, as evidenced by no appreciable reduction in the pericardial effusion compared to the scan from March 6, 2025. The patient was alive and under continued follow-up as of August 2025.

## 5 Discussion

FO-LBCL primarily affects elderly patients, with a median age of 70 years; over 95% of cases occur in individuals aged 60 or older. Most patients have no underlying immunodeficiency. The disease is frequently associated with conditions that cause fluid overload, including chronic heart failure, chronic renal failure, liver cirrhosis, and protein-losing enteropathy (36). EBV co-infection is present in only 9% to 29% of cases. Crucially, all cases are negative for KSHV/HHV-8, a key finding that helps differentiate FO-LBCL from PEL (24, 41). The clinical presentation is predominantly characterized by serous cavity effusions. The pleural cavity is most frequently involved (76%), followed by the pericardial (38%) and peritoneal (12%) cavities. A notable geographic variation exists, with Japanese patients demonstrating a significantly higher rate of pericardial involvement than non-Japanese patients (56% vs. 24%, p< 0.0001). Approximately 40% of patients present with effusions in multiple cavities simultaneously. The most common initial symptom is dyspnea, occurring in two-thirds of patients. This is often accompanied by lower extremity edema and, in some cases, B symptoms. Simultaneous involvement of multiple serous cavities is observed in approximately 40% of patients. The most common initial symptom is dyspnea, presenting in two-thirds of cases and often accompanied by lower limb edema. Less commonly, patients may experience B symptoms, including fever, night sweats, and weight loss (42, 43). This study’s analysis of 57 patients aligns with previous reports, confirming consistent patterns in the sites of involvement and initial clinical manifestations. Furthermore, neither of the two patients presented in this case series showed evidence of infection with KSHV/HHV-8 or EBV. The etiology of FO-LBCL remains unclear, though the retention of body cavity effusions is a proposed contributing factor (36); Additional studies suggest an association between FO-LBCL and HCV infection, with reported infection rates of 22% to 25% (24, 41), HCV may function as a persistent antigenic stimulus, driving the clonal expansion of peritoneal B-cells. This prolonged proliferation provides a context for the accumulation of additional genetic alterations, such as the t(8;22) translocation, ultimately culminating in the development of FO-LBCL (44, 45).

The tumor cells of FO-LBCL are characterized as medium to large lymphocytes with abundant, often vacuolated, basophilic cytoplasm. The nuclei are pleomorphic, exhibiting round, oval, or irregular contours, and contain prominent single or multiple eosinophilic nucleoli. The chromatin is loosely distributed and may appear vacuolated. Nuclear invaginations resulting in lobulated or binucleated forms are common. These cells display high mitotic activity and frequent apoptotic bodies, with morphological features that can range from centroblastic to immunoblastic or plasmablastic (24, 42, 43). The immunophenotype of FO-LBCL is characterized by consistent expression of pan-B-cell markers, including CD20 (98%), PAX5 (100%), and CD19. In contrast, markers such as CD138, CD45, and CD3 are typically absent. Molecular profiling most frequently identifies the non-germinal center B-cell subtype. Immunohistochemistry reveals high BCL2 expression (73%) and heterogeneous MYC expression (34%), with approximately 30% of cases demonstrating co-expression of MYC and BCL2 (43); Cases with unusual immunophenotypes have also been reported. These include instances lacking conventional pan-B-cell markers despite demonstrating clonal B-cell gene rearrangements (46), or those exhibiting an aberrant T-cell phenotype (47). Molecular genetic analyses revealed rearrangement rates of 19% (20/107) for *MYC*, 29% (16/55) for *BCL6*, and 11% (6/54) for *BCL2*. Furthermore, approximately 50% of cases displayed complex karyotype abnormalities, including the t(9;14) translocation (36, 43). The two reported cases exhibited consistent tumor morphology and immunophenotype. However, flow cytometry of the second patient’s pleural fluid revealed an absence of CD19 expression. This finding aligns with the broader literature, wherein this review identifies CD20 negativity in 13.7% of patients and CD138 positivity in 30.4%. These immunophenotypic variabilities underscore that a definitive diagnosis cannot rely on a limited panel of markers and must instead integrate comprehensive morphological, immunophenotypic, and clinical data to prevent misdiagnosis.

Serosal lesions in patients with FO-LBCL typically demonstrate intense fluorodeoxyglucose (^18^F-FDG) avidity on PET-CT. However, this hypermetabolism is non-specific and can be observed in other conditions involving the serosa, such as metastatic malignancies, other hematologic neoplasms, or inflammatory diseases. Consequently, a key utility of PET-CT in this context is to exclude other space-occupying pathologies within the serosal cavities. Furthermore, PET-CT offers superior delineation of diffuse serosal infiltration, which is often poorly defined on conventional CT, thereby enhancing its value for initial staging and post-therapeutic response assessment. Despite its higher cost and the currently limited clinical experience specific to FO-LBCL, we recommend PET-CT for suspected or confirmed cases to aid in comprehensive differential diagnosis, accurate staging, and subsequent monitoring of treatment efficacy.

PEL and FO-LBCL share highly similar clinical presentations, rendering them indistinguishable based solely on clinical features, routine laboratory tests, or bone marrow morphology. PEL is a distinct subtype of non-Hodgkin lymphoma defined by malignant effusions in body cavities. It predominantly affects middle-aged and young men (median age ~40 years), with a strong association with immunocompromised states; approximately 80% of cases occur in HIV-positive individuals (24). According to the WHO classification, PEL is defined as a large B-cell lymphoma that typically lacks expression of pan-B-cell markers (e.g., CD19, CD20, CD79a) although it often expresses CD45, CD138, MUM1, and EMA, and may aberrantly express T-cell markers such as CD3 and CD4 (48); A definitive diagnosis of PEL requires conclusive evidence of KSHV/HHV-8 infection, which is present in all cases; approximately 60%–80% of these are co-infected with EBV. In contrast to some other large B-cell lymphomas, PEL is not associated with *c-myc* gene rearrangements, a critical distinguishing feature (49, 50). Based on the absence of KSHV/HHV-8 infection and the presence of B-cell marker expression, the cases in this report were inconsistent with a PEL diagnosis (49, 51). Based on the absence of KSHV/HHV-8 infection and the presence of B-cell marker expression, the cases in this report were inconsistent with a PEL diagnosis.

Additionally, FO-LBCL must be differentiated from pyothorax-associated lymphoma (PAL) and diffuse large B-cell lymphoma with malignant effusion. ① PAL: Although both PAL and FO-LBCL are rare large B-cell lymphomas originating in body cavities, they are distinguished by key clinical and pathological features. PAL predominantly affects elderly patients with a long-term history of chronic empyema or pleurisy and is associated with EBV infection in approximately 85% of cases, usually in immunocompetent individuals. In contrast, FO-LBCL typically arises without this specific inflammatory history and shows no strong association with EBV. Pathologically, while both entities express B-cell markers such as CD20, aberrant expression of T-cell markers (e.g., CD2, CD3, CD4) can be found in approximately 30% of PAL cases. Furthermore, PAL is characterized radiologically by an eccentric, lens- or crescent-shaped soft tissue mass within the empyema cavity (50, 52, 53); ② Diffuse large B-cell lymphoma (DLBCL) with malignant effusion: Although the tumor cells of conventional DLBCL with malignant effusion and FO-LBCL can exhibit overlapping morphological and immunophenotypic features, their underlying etiologies are fundamentally distinct. Conventional DLBCL typically manifests with primary solid lesions, such as lymphadenopathy or extranodal masses; associated effusions are secondary phenomena resulting from advanced-stage dissemination or local infiltration. In contrast, FO-LBCL is primarily defined by the effusion itself, often in the absence of a dominant tumor mass. Consequently, the differential diagnosis critically relies on imaging techniques, particularly PET-CT, to evaluate the systemic disease burden and identify or rule out a primary solid lesion (54). Other lymphomas that may present with malignant body cavity effusions—including Burkitt lymphoma, mantle cell lymphoma, anaplastic large cell lymphoma, and peripheral T-cell lymphoma—must be considered in the differential diagnosis. These entities can be definitively distinguished from FO-LBCL based on their distinct clinical presentations, histopathological features, and immunophenotypic profiles.

Due to its rarity, no unified treatment standard has been established for FO-LBCL. Despite exhibiting the histopathological characteristics of a large B-cell lymphoma, FO-LBCL typically follows a more favorable clinical course than conventional DLBCL, with some cases demonstrating an indolent progression. The indolent nature of FO-LBCL is further evidenced by cases diagnosed only after six years of continuous monitoring for pericardial effusion (55), as well as by patients who have achieved prolonged, treatment-free remission—exceeding 18 months—even after experiencing recurrence and undergoing multi-drug salvage chemotherapy (56); A subset of patients can achieve complete remission with paracentesis alone, without systemic chemotherapy. In the present cohort, 43.9% of patients were managed without chemotherapy. Among those with available follow-up data, the survival rate for this non-chemotherapy group was only marginally lower than that of patients who received chemotherapy (61.2% vs. 70.0%). One hypothesis for this phenomenon is that paracentesis may lead to the release of tumor antigens, potentially stimulating a local anti-tumor immune response; however, this mechanism remains unconfirmed (22, 57, 58). Current evidence supports anthracycline-based chemotherapy as the first-line treatment for FO-LBCL, with studies indicating favorable outcomes. For instance, Kaji (42) et al. reported that among 56 Japanese patients treated with the CHOP or R-CHOP regimen, the overall response rate was 95%, with a complete remission rate of 73% and a 2-year overall survival rate of 84.7%. Furthermore, a larger analysis by Gisriel (43) et al. of 202 patients identified key prognostic factors. Residence in Japan was associated with a trend toward improved survival (hazard ratio [HR] = 0.475; 95% confidence interval [CI], 0.216–1.044; p = 0.064), while the presence of peritoneal effusion was an independent adverse prognostic factor (HR = 3.652; 95% CI, 1.763–7.565; p = 0.004).

These findings are supported by an independent review, which identified Japanese ethnicity, pericardial effusion, CD20 positivity, and rituximab-containing chemotherapy as favorable prognostic factors, while peritoneal effusion and CD20 negativity were unfavorable. Consistent with this, the present study also confirms CD20 positivity and rituximab-based chemotherapy as predictors of a favorable outcome. Furthermore, our analysis identified additional favorable prognostic factors, including CD79a positivity, CD138 negativity, age greater than 65 years, and a lactate dehydrogenase (LDH) level ≤ 500 U/L. In contrast to previous reports, our study identified pleural effusion as a favorable prognostic factor. This discrepancy is likely attributable to our study’s limited cohort size. Consequently, clinical prognosis should not be determined by the site of involvement alone but through a multifactorial assessment. Multivariate analysis confirmed that age >70 years and CD20 positivity were independent favorable prognostic factors. Notably, patients with CD20-positive FO-LBCL exhibited significantly improved outcomes following rituximab-containing therapy. These findings suggest that rituximab-based chemotherapy represents an appropriate first-line standard of care for this patient subgroup (59). For patients who are candidates for systemic chemotherapy, CHOP-based regimens constitute the cornerstone of treatment. However, for elderly patients or those with significant comorbidities that preclude chemotherapy, management may be limited to the drainage of effusions (43).

A 72-year-old male with FO-LBCL demonstrated only 10% CD20 positivity on ascites pathology. This low level of expression was directly associated with his resistance to CHOP chemotherapy and poor prognosis (5). The low expression or loss of CD20 is frequently observed in FO-LBCL and may arise from the intrinsic biology of the tumor cells or from clonal selection induced by therapeutic pressure. This phenomenon is documented in prior case reports; for instance, one described a patient with relapsed FO-LBCL whose immunophenotype shifted from CD20-positive at diagnosis to CD20-negative at relapse, with concomitant loss of other markers including CD79a and MUM1. These alterations in antigen expression significantly compromise the efficacy of R-CHOP-based chemotherapy (10).

This study demonstrates a 80.0% prevalence of CD79a expression in FO-LBCL. CD79a and CD79b form the heterodimeric signaling component of the B-cell receptor, with CD79b being nearly universally expressed in mature B-cell lymphomas and normal B cells (60). This expression profile makes CD79b a compelling therapeutic target. Polatuzumab vedotin, an antibody-drug conjugate directed against CD79b, received U.S. FDA approval in June 2019 in combination with bendamustine and rituximab for patients with relapsed/refractory DLBCL after at least two prior therapies (61). More recently, polatuzumab vedotin has shown superior efficacy to standard R-CHOP in frontline therapy. The phase III POLARIX trial (NCT03274492) compared Pola-R-CHP with R-CHOP in previously untreated, high-risk DLBCL patients, demonstrating a statistically significant improvement in progression-free survival (PFS) for the polatuzumab-containing regimen (2-year PFS 76.7% vs. 70.2%) and a significant reduction in the risk of disease progression, relapse, or death (62). Although FO-LBCL was not included in this trial, its typically aggressive clinical behavior and variable response to conventional chemotherapy parallel the high-risk profiles of the enrolled patients. Given the high frequency of CD79a/b complex expression in FO-LBCL, CD79b represents a rational therapeutic target for this subtype. We recommend the routine assessment of CD79b expression in FO-LBCL cases and advocate for future clinical trials to evaluate the efficacy of polatuzumab vedotin in this and other rare lymphoma entities.

In precision medicine, elucidating the molecular basis of FO-LBCL is critical for developing targeted therapies. Although FO-LBCL exhibits distinct clinical and immunophenotypic features, our study and prior literature suggest the absence of a unifying genomic driver. Instead, its clinical peculiarity may arise from epigenomic dysregulation. Epigenetic mechanisms are pivotal in germinal center B-cell differentiation and are frequently disrupted in aggressive lymphomas, leading to differentiation arrest, immune evasion, and therapy resistance. Supporting this, Pascual (7) et al. identified pathogenic or likely pathogenic mutations in the epigenetic regulators *KMT2D* and *EP300* in FO-LBCL patients. Loss-of-function mutations in *KMT2D*, which encodes an H3K4 methyltransferase, diminish enhancer activity and repress differentiation-related genes, thereby maintaining tumor cells in an undifferentiated state. Similarly, dysfunction of the histone acetyltransferase *EP300* impairs chromatin-mediated transcriptional activation and immune signaling (63). Given the demonstrated potential of epigenetic therapies in B-cell lymphomas, future research should leverage multi-omics approaches to define epigenetic subtypes of FO-LBCL. This strategy promises to uncover prognostic and predictive biomarkers, reveal novel targets for personalized therapy, and establish epigenetics as a cornerstone for future molecular classification and targeted treatment.

In summary, FO-LBCL is characterized by its primary manifestation as serous cavity effusion in the absence of solid masses, its occurrence in immunocompetent elderly patients with cardiorenal or hepatic comorbidities, and its distinct pathological profile, which is not associated with KSHV/HHV-8 infection. This review synthesizes the distinct clinicopathological features of FO-LBCL to aid clinicians in its diagnosis and differential diagnosis. A high index of suspicion is warranted when a patient’s serous cavity effusion is unexplained by known comorbidities, even in the setting of normal routine hematologic parameters. In such cases, early cytopathological and immunohistochemical analysis of the effusion is critical for a definitive diagnosis. Furthermore, this article outlines principles for prognostic assessment and treatment selection, emphasizing that a comprehensive evaluation is essential for staging, determining prognosis, and formulating an individualized management plan.

## Data Availability

The original contributions presented in the study are included in the article/supplementary material. Further inquiries can be directed to the corresponding authors.
